# Baicalein inhibits the progression of thyroid cancer by suppressing the TPL2/MEK2/ERK2 pathway

**DOI:** 10.3389/fendo.2026.1739944

**Published:** 2026-01-28

**Authors:** Nan Wu, Yang Wu, Qian Zhang, Muhammad Naeem, Ren Jing, Yuan-bin Luo, Shijian Yi

**Affiliations:** 1 Department of Breast and Thyroid Surgery, South China Hospital, Medical School, Shenzhen University, Shenzhen, China; 2 Respiratory Medicine, Shenzhen Pingle Orthopedic Hospital, Shenzhen, China

**Keywords:** baicalein, Golgi apparatus, MAPK pathway, PLAU, PTC, thyroid cancer

## Abstract

**Introduction:**

Papillary thyroid cancer (PTC) is the most common type of endocrine malignancy caused by genetic mutations, hormonal imbalances, and environmental factors. However, recurrent infections, and metastasis in PTC patients remain challenged due to complexity of traditional methods. Baicalein (BA) is a kind of natural flavonoid that exhibits the anti-cancer, anti-inflammatory, anti-tumor, and anti-viral activities. The molecular mechanism of baicalein in pathogenesis of PTC remains unclear. This study was designed to explore the inhibitory effects of BA against PTC by mediating the Golgi apparatus reprogramming via PLAU and suppressing the TPL2/MEK2/ERK2 pathway.

**Methods:**

Transcriptomic analysis was performed to explore the gene expression profiles. Molecular docking was employed to identify the potential targets to elucidate the molecular mechanism of action of BA.

**Results:**

PLAU, an up-regulated DEG, is implicated in tumor development, lymph node metastasis, and infiltration levels of neutrophils and dendritic cells in thyroid cancer patients. Molecular docking analysis revealed that serum levels of uPA protein encoded by PLAU and *Plau* mRNA were elevated in PTC patients with metastasis and BRAF mutation. BA treatment upregulates *PLAU* gene expression, but this increased PLAU protein subsequently interacts with and inhibited by BA, leading to downstream pathway suppression.

**Conclusion:**

It was concluded it could be served as a promising therapeutic strategy for the treatment of PTC.

## Graphical Abstract

**Figure:**
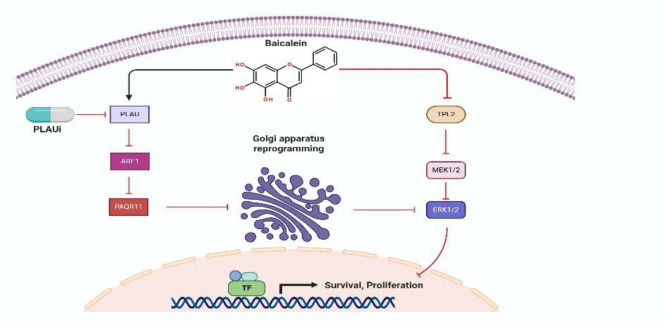

## Introduction

Thyroid cancer is the type of malignant tumor in the endocrine system, ranking ninth cancer in the global incidence with mortality rate (1–3). Papillary thyroid carcinoma (PTC) constitutes about 84% of all the thyroid cancer pathological types. PTC patients exhibited the poor survival rate due to distant metastasis, complicated diagnosis, recurrent infections, and poor clinical management. The recurrent laryngeal nerve paralysis (RLNP) resulting in high invasion rate (1, 4). Current treatment options for papillary thyroid cancer are limited to surgery, targeted therapies, and immunotherapy. These treatment options are ineffective due to some limitations. Chemoradiotherapy can cause the significant toxicity issues, targeted therapies frequently increased the drug resistance, and immunotherapy has shown limited efficacy (5). These challenges can be overcome through discovering the novel therapeutic agent with high specificity to prevent pathogenesis of papillary thyroid carcinoma (6, 7).

In thyroid cancer, *PLAU* is often overexpressed and contributes to tumor invasion and metastasis by degrading the extracellular matrix. It also promotes cancer cell invasion, migration, and metastasis through activation of proteolytic pathways (8–10). As a molecular chaperone, *PLAU* promotes the activity of class III phosphatidylinositol 3-kinase (PI3K) near damaged mitochondria, with disruption of the mitochondrial autophagic flux leading to the ROS-mediated apoptosis. PLAU also mediated the mitochondrial biogenesis through the PPARGC1A/PGC1α pathway, maintaining the mitochondrial homeostasis. However, the role of PLAU in PTC dedifferentiation remains unexplored. Therefore, investigating the molecular mechanisms in BA and PLAU in PTC cells is crucial in signaling pathway influenced the thyroid cancer (11, 12).

Baicalein is a kind of natural flavonoid derived from the medicinal plant *Scutellaria baicalensis*. Recent studies showed that BA exhibited the different pharmacological activities anti-tumor, anti-inflammatory, antioxidant, and hepatoprotective (13–15). BA has also anti-tumor effects on TC, inhibiting the ERK1/2 and PI3K/Akt pathways to induce the apoptosis and autophagy in TC cells (16, 17). Our previous research suggested that BA activates the NF-κB signaling pathway to induce the autophagy and apoptosis, while inhibiting the mitotic protein cyclin B1 to cause mitotic cycle arrest in TC cells (18). However, the potential molecular mechanisms of BA in PTC have not yet been fully investigated (15).

This study demonstrated the inhibitory effects of BA on PTC by suppressing the MEK-ERK pathway and mediated the Golgi apparatus reprogramming through the PLAU activation. Protein expression was performed through the western blot analysis. Molecular docking, and transcriptomics analysis were performed for gene expression profiles, and identification of potential targets to elucidate molecular mechanism of action of BA against thyroid cancer. The findings of this study could be helpful in the management of papillary thyroid cancer.

## Methods

### Chemicals and reagents

Baicalein (BA) with a purity of ≥98% (HPLC) was obtained from the Medical Chem Express Co., Ltd (HY-N0159, Shanghai, China). BC-11 hydrobromide, a PLAU inhibitor (PLAUi), was obtained from Tocris, Biotechnology (Bristol, UK). Dulbecco’s modified eagle’s medium (DMEM, 11995065) and fetal bovine serum (FBS, 10099141) were obtained from Gibco (Grand Island, NY, USA) and Invitrogen (Life Technologies, Carlsbad, CA, USA), respectively. Additionally, Golgi apparatus-tracker red (C1043) and Mito-tacker green (C1048) were purchased from the Beyotime Biotechnology Co., Ltd. Anti-GAPDH antibody (5174S) was purchased from Cell Signaling Technology, Ltd. (Beverly, USA). Human PTC cell lines (KTC-1) were purchased from the Zhong Qiaoxinzhou Biotechnology Co., Ltd. (Shanghai, China). PrimeScript™ RT reagent Kit with gDNA Eraser (Perfect Real Time) (RR047A) and TB Green^®^ Premix Ex Taq™ (Tli RNaseH Plus) (RR420A) for the qPCR were purchased from Takara Biotechnology Co., Ltd. (Beijing, China).

### Samples collection and tissue culture

Six pairs of human PTC tissues both with or without lymph node or distant metastasis, six human PTC tissues harboring the *BRAF^V600E^* mutation were obtained from South China Hospital, Shenzhen University in accordance with institutional guidelines. None of the patients had undergone the radioactive iodine therapy or radiofrequency ablation prior to surgical resection. Firstly, collected samples cells were cultured in DMEM supplemented with 0.1% FBS and 1% streptomycin and penicillin (B21210; R&D systems, USA) and incubated at 37°C with 5% CO_2_.

### RNA extraction and quantitative real-time PCR

Total RNA was extracted from tumor tissues and cultured KTC-1 cells using Trizol™ reagent (Carlsbad, CA, USA) following the protocol provided by the manufacturer. Complementary DNA was then synthesized employing the PrimeScript™ RT reagent Kit with gDNA Eraser (Perfect Real Time). Subsequently, RT-qPCR analysis was performed by utilizing the TB Green^®^ Premix Ex Taq™ II (Tli RNaseH Plus) on a qTOWER384G fluorescence RT-qPCR instrument (Analytik Jena AG, Jena, Germany). RT-qPCR primers used in this study were shown in **Table 1**. Each sample underwent triplicate analysis to quantitatively assess RNA amplification. The relative gene expressions were normalized against GAPDH within each sample, using the comparative threshold cycle method (2^-△△CT^ method).

**Table 1:** Shows the list of qPCR primers used in this study.

Gene	Synonyms	Direction	Size (bp)	Sequence (5’→3’)
PLAU	uPA	Forward	127	CGCTCAAGGCTTAACTCCAACAC
		Reverse	127	AACGGATCTTCAGCAAGGCAATG
ERK1	MAPK3	Forward	127	CATTGTGCAGGACCTGATGGAGAC
		Reverse	127	GTTGGCGGAGTGGATGTACTTGAG
ERK2	MAPK1	Forward	101	TCGCCGAAGCACCATTCAAGTTC
		Reverse	101	TCCTGGCTGGAATCTAGCAGTCTC
MEK1	MAP2K1	Forward	114	TCATCTGGAGATCAAACCCGCAATC
		Reverse	114	CCATCGCTGTAGAACGCACCATAG
MEK2	MAP2K2	Forward	124	CGCTCACCATCAACCCTACCATC
		Reverse	124	TTCTTCTGCTGCTCGTCAAGTTCC
TPL2	MAP3K8	Forward	91	GCACAGGAAGCACCGAGGAATC
		Reverse	91	ACAAGATTGAAGTAGCCAGCCAGAG
NIS	SLC5A5	Forward	96	CTGCTGGTGCTGGACATCTTCG
		Reverse	96	GCTGGTGGATGCTGTGCTGAG
ARF1		Forward	145	GAACATCTTCGCCAACCTCTTCAAG
		Reverse	145	GCCTATGGTGGGAATGGTGGTC
MET		Forward	106	AACAACACCGATGGACTTCAGGAAC
		Reverse	106	AGATGGCGTGGGCAAATGGAAT
GAPDH		Forward	115	GGCACAGTCAAGGCTGAGAAT G
		Reverse	115	ATGGTGGTGAGACGCCAGTA

### RNA sequencing analysis

The integrity of RNA was evaluated through the Bioanalyzer 2100 system (Agilent Technologies, CA, USA). Total RNA served as the input material for the RNA sample preparations and mRNA was purified from the total RNA using Poly-T oligo-attached magnetic beads. The purified mRNA was reverse transcribed into first strand cDNA with random hexamer primer and M-MuLV reverse transcriptase (RNase H-). The second strand of cDNA was synthesized using the DNA Polymerase I and RNase H. Exonuclease/polymerase activities were used to convert the remaining overhangs into blunt ends. Following the adenylation of the 3’ ends of DNA fragments, adaptors featuring a hairpin loop structure were ligated to prepare them for hybridization. To select cDNA fragments with a preferred length of 370–420 bp, library fragments were purified using AMPure XP system. The PCR products were purified using the AMPure XP system and the library quality was assessed on the Agilent Bioanalyzer 2100 system. The quality of the mRNA sequencing data was evaluated using the FastQC method.

### Differential genes expression analysis

The index-coded samples were clustered using the cBot cluster generation system that combined features with the TruSeq PE Cluster Kit v3-cBot-HS (Illumia) according to the manufacturer’s instructions. Following the generation of clusters and gene abundance analysis, library preparations were also sequenced on an Illumina Novaseq platform, resulting in 150 bp paired end reads. Differential expression analysis was conducted for the identification of DEGs (Differentially Expressed Genes) between pairwise comparisons, and also quantifying the gene expression based on FPKM values (fragments per kilobase of transcript sequence per million mapped reads). DEGs from KTC-1 cells were screened in each comparison, with a threshold of |log2 fold change (FC)| ≥ 2.0 and *P* < 0.01. The top 5 up-regulated and down-regulated DEGs from each comparison were ranked using the RobustRankAggreg package in R software. These DEGs were then utilized to construct the protein-protein interaction (PPI) network through the STRING (version 10.5) database. Subsequently, Cytoscape software’s cytoHubba plugin was employed to identify significant hub DEGs from the PPI network, with default parameters and based on the maximal clique centrality (MCC) and eleven other computing methods. Finally, RobustRankAggreg package core algorithm was applied once more to rank the hub DEGs, considering results from the aforementioned 12 computing methods, with criteria of Frequencies ≥ 12 and Score < 0.05 as statistically significant.

Gene Ontology (GO) and the Kyoto Encyclopedia of Genes and Genomes (KEGG) pathway enrichment analyses were conducted on the hub DEGs using the DAVID (version 6.8) database (https://davidbioinformatics.nih.gov/). Moreover, the expression patterns and survival outcomes of the hub DEGs were investigated through the GEPIA, UALCAN, and Human Protein Atlas (HPA) databases. Furthermore, the association of hub DEGs with immune function and the infiltration levels of various immune cells was assessed using the tumor immune estimation resource database (TIMER).

### Experimental mice model

Four-week-old nude mice were housed in clean pathogen-free conditions. After a one-week acclimatization period, mice were subcutaneously injected with KTC-1 cells (1× 10^6) to establish the xenograft model. Tumor growth was monitored by measuring tumor volumes and weights at intervals of every three days. Tumor volume was calculated using the formula V = (width)^2 * length/2. When the tumor volumes ranged from 50 to 100 mm^3, the mice (*n* = 18) were randomly assigned into one of three groups: control, BA, and BA combined with PLAUi, with 6 mice per group. The BA groups were received a daily dose of 100 mg/kg of BA (19), and BA+PLAUi groups were administered same dose of BA in combination with BC-11 (final concentration 5 mM). Cyclophilin B (PPIB) expression can vary under stress. In our specific model (KTC-1 cells and xenografts under the tested conditions, PPIB demonstrated the stable expression across all treatment groups in our validation assays (15, 19).

### Cell viability assay

To elucidate the alterations in gene expression and transcriptome profiles, KTC-1 cells were subjected to treatment with BA at concentrations of 0, 50, 100, and 200 μM for a duration of 24 h. For *in vitro* experiments, KTC-1 cells were randomly assigned to one of four groups: Ctrl, BA (100 μM), PLAUi (10 μM), and a combination of BA (100 μM) with PLAUi (10 μM) (15, 19). These cells were seeded into 96-well plates at a density of 1× 10^5 cells per well and incubated for 24 h at 37°C in an atmosphere of 5% CO_2_. Cell viability was assessed using CCK-8 assay, following the guidelines provided by the manufacturer. The absorbance values were obtained at a wavelength of 450 nm using a microplate reader (Thermo Fisher, USA).

### Wound healing assay

KTC-1 cells were seeded into six-well plates and vertical incision was then introduced at center of each well using a pipette tip with a capacity of 1.0 ml. Subsequently, plates underwent three rounds of washing to eliminate the dislodged cells. The cells were treated with control, BA (100 μM), PLAUi (10 μM), and a combination of BA (100 μM) with PLAUi (10 μM). The impact of BA or PLAUi on migration of KTC-1 cells was monitored under a microscope at intervals of 24 h and 48 h by quantifying the alterations in scratch width. The scratch healing rate was calculated by subtracting scratch width at a given time point from initial scratch width, dividing this difference by the initial scratch width, and then multiplying by 100%.

### Apoptosis analysis

The apoptosis detection kit was employed to evaluate the effect of BA or PLAUi on KTC-1 cells. Following the administration of drugs, the cells were harvested in 100 μl of 1X Annexin V binding buffer. The cell suspension was subsequently treated with 2.5 μl of Annexin V-FITC and 2.5 μl of PI solution and then incubated on ice for 20 min in the absence of light. Subsequently, the sample was gently mixed with an extra 400 μl of 1X Annexin V binding buffer prior to flow cytometry and fluorescence microscopy analyses.

### Mitochondria and Golgi apparatus staining

To visualize the mitochondria and Golgi apparatus in living KTC-1 cells, cells treated with various drugs were separately incubated in medium containing Mito-Tracker or Golgi apparatus-Tracker at 37°C for 30 min. Subsequently, cells were stained with Hoechst 33342 for 5 minutes and examined using the multiplex confocal microscopy (LSM980, Zeiss, Oberkochen, Germany).

### Western blotting

Protein extraction was carried out using the RIPA lysis buffer (20-188; Sigma-Aldrich, USA), supplemented with a Protease Inhibitor Tablet (11836170001; Roche, Switzerland) and a PhosphoSTOP Phosphatase Inhibitor Tablet (4906845001; Roche, Switzerland). The protein extracts were subjected to SDS-PAGE electrophoresis, followed by transfer onto polyvinylidene fluoride (PVDF) membranes. After blocking with 5% skimmed milk, the membranes were incubated with primary antibodies, followed by the corresponding secondary antibodies. The densities of the protein bands were quantified from scanned images using BeyoECL Plus reagent (P0018S; Beyotime, Shanghai, China).

### Statistical analysis

Data were processed by using the GraphPad Prism 9 (San Diego, CA, USA). The normality of data was assessed using the Shapiro-Wilk test. Data that were normally distributed are presented as means ± S.E.M. To compare two groups, the two-tailed Student’s T-test was employed, whereas for multiple groups, one-way ANOVAs were conducted, followed by Tukey’s multiple comparisons test. The two-way ANOVAs with Bonferroni correction was performed for comparing multiple groups across various time points. The *p*-value of less than 0.05 was considered statistically significant.

## Results

### Single-cell transcriptomics analysis

**Figure 1:**
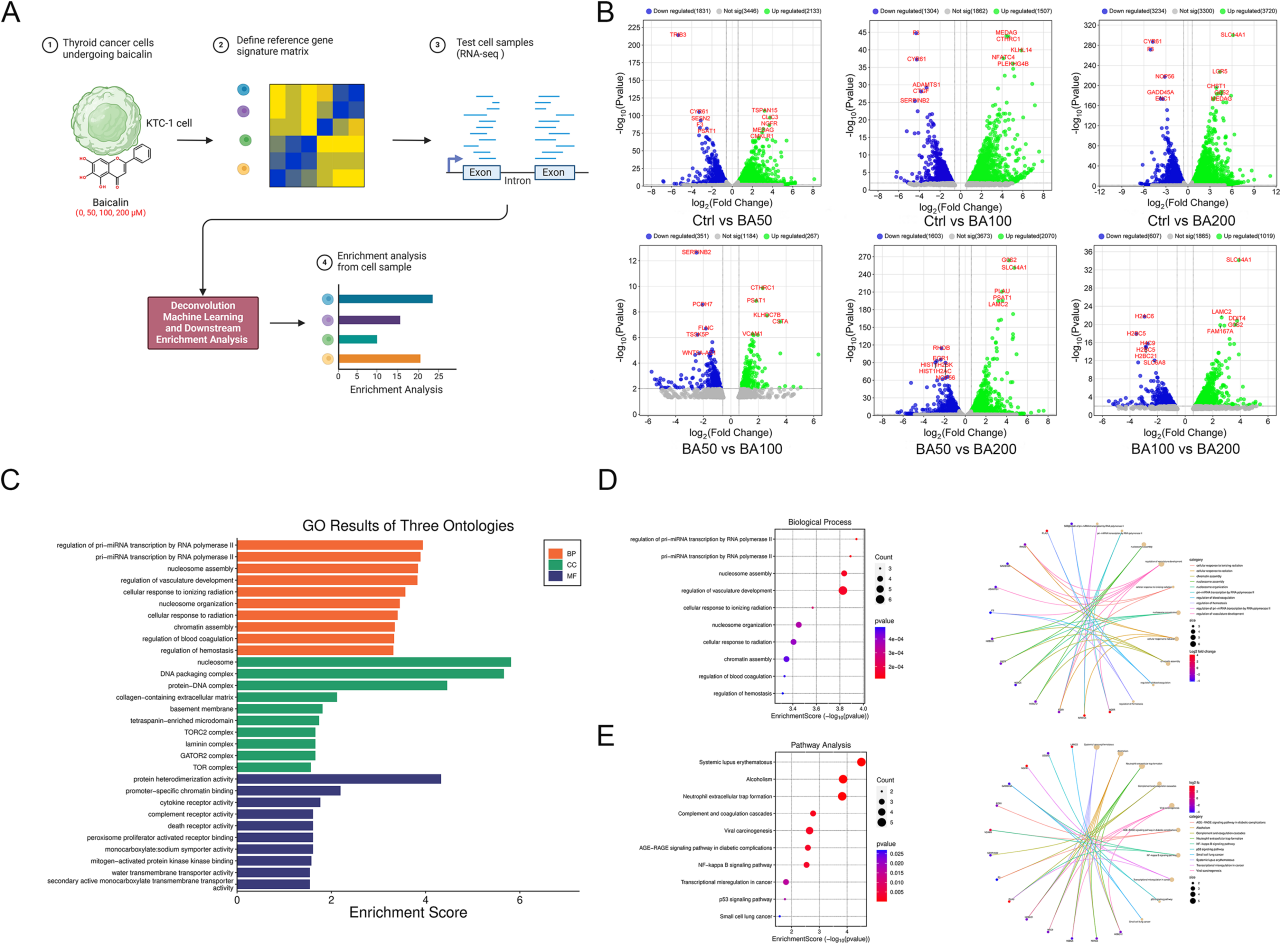
Identification of differentially expressed genes and enrichment analysis in baicalein-treated KTC-1 Cells. **(A)** Overview of the study design and the RNA-sequence workflow. **(B)** Identification of differentially expressed genes, with the top 5 up-regulated and top 5 down-regulated genes in each pairwise comparison visualized using a Volcano plot. **(C)** Gene Ontology (GO) enrichment analysis encompassing biological process, cellular component, and molecular function, derived from a gene list comprising the top 5 up-regulated and top 5 down-regulated genes from each pairwise comparison. **(D)** Depiction of a representative biological process and its corresponding differentially expressed genes. **(E)** Kyoto Encyclopedia of Genes and Genomes (KEGG) pathway enrichment analysis conducted using a gene list compiled from the top 5 up-regulated and top 5 down-regulated genes from each pairwise comparison. The abbreviations Ctrl, BA50, BA100, and BA200 respectively denote KTC-1 cells treated with 0, 50, 100, and 200 μM of baicalein.

To investigate the molecular mechanism underlying the inhibition of TC cells by BA, KTC-1 cells were treated with varying concentrations of BA (0, 50, 100, 200 μM) (**Figure 1A**). Substantial number of DEGs were identified in each pairwise comparison with top 5 up-regulated, and top 5 down-regulated DEGs highlighted (**Figure 1B**). GO analysis indicated the significant enrichment of genes associated with pri-miRNA transcription, nucleosome assembly, DNA packaging, protein and heterodimerization activity (**Figures 1C, D**). These genes were found to be enriched in various KEGG pathways, including systemic lupus erythematosus, neutrophil extracellular trap formation, NF-κB signaling pathway, and transcriptional dysregulation in cancer (**Figure 1E**).

Furthermore, gene set enrichment analysis (GSEA) and pathway enrichment revealed that numerous pathways were positively associated with BA-induced inhibition of KTC-1 cells. In comparison to control group, BA50 suppressed the MYC targets and unfolded protein response, whereas BA100 enhanced the interferon-γ response but restricted MYC targets. Conversely, BA200 stimulated the interferon-α response and suppressed the MYC targets. BA100 was found to promote both interferon-α and interferon-γ response as well as spermatogenesis, whereas BA200 stimulated the E2F targets, interferon-α response and the epithelial mesenchymal transition. Moreover, BA200 facilitated the apical junction and mitotic spindle formation but inhibited oxidative phosphorylation relative to BA100 (**Supplementary Figure S1A**). Within the comparison between the control and BA-treated cells, DEGs were enriched in pathways related to extracellular stimulus response, extracellular structure and matrix organization. DEGs between BA50 and BA100 were involved in amino acid biosynthetic and metabolic process, while DEGs from the comparisons between BA50 and BA200, as well as BA100 and BA200, were also enriched in pathways related to extracellular stimulus response, extracellular structure and matrix organization (**Supplementary Figure S1B**).

### PLAU role in tumor development

**Figure 2:**
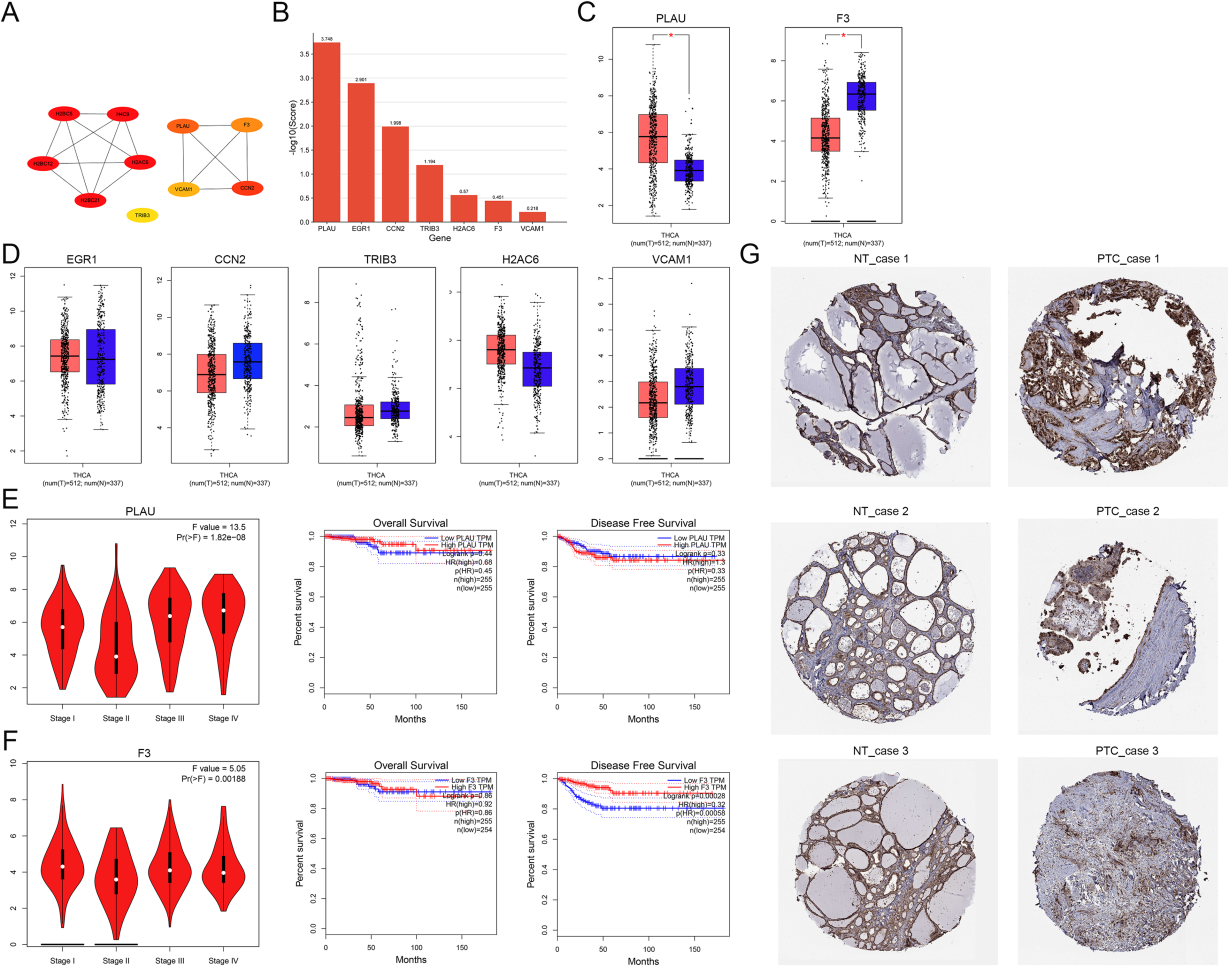
Identification and annotation analysis of hub differentially expressed genes in baicalein-treated KTC-1 cells. **(A)** Protein-protein interaction network of hub differentially expressed genes all BA-treated groups (50, 100, 150 μM) versus the control group. **(B)** Hierarchical clustering of hub differentially expressed genes using the *RobustRankAggreg* method. **(C)** Boxplot illustrates the significant differences in PLAU and F3 mRNA levels between thyroid carcinoma and matched normal thyroid samples. **(D)** Boxplot showing the mRNA levels of EGR1, CCN2, TRIB3, H2AC6, and VCAM1 in thyroid carcinoma compared to matched normal thyroid samples, with no significant differences observed. **(E)** PLAU expression correlates with tumor stage, but it is not linked to overall survival and disease-free survival. **(F)** F3 expression correlates with tumor stage, overall survival, and disease-free survival. **(G)** Immunohistochemical analysis of PLAU expression in papillary thyroid carcinoma versus normal thyroid tissues from the HPA database. *p < 0.05.

The PPI network was constructed as shown in **Figure 2A**. The comprehensive analysis of data from the twelve computational methods by cytoHubba revealed that PLAU, EGR1, CCN2, TRIB3, H2AC6, F3, and VCAM1 were identified as central DEGs, exhibiting statistical difference (**Figure 2B**, **Supplementary Table S1**). The TCGA database indicated that PLAU was up-regulated and F3 was down-regulated in TC tumor tissues compared to normal tissues (**Figure 2C**, **Supplementary Figure S2A**). However, the expressions of EGR1, CCN2, TRIB3, H2AC6, and VCAM1 were not displayed statistically significant differences between NT and TC tissues (**Figure 2D**). PLAU expression was not found to correlate with gender, race, and age of TC patients (**Supplementary Figure S2B**). Patients with stage 3 or stage 4 of TC exhibited higher PLAU expression as compared to stage 1 (**Supplementary Figure S2C**). The PLAU expression in follicular PTC was reduced relative to classical PTC (**Supplementary Figure S2D**). Furthermore, PLAU expression positively correlated with lymph node metastasis (**Supplementary Figure S2E**). Nevertheless, PLAU expression did not correlate with overall survival and disease-free survival of TC patients (**Figure 2E**). F3 expression was also linked to tumor stage, with low F3 expression in TC patients associated with poor disease-free survival (**Figure 2F**). The HPA database showed that PLAU expression in PTC patients was significantly higher than NT from healthy individuals (**Figure 2G**). These findings suggested that PLAU is up-regulated DEG implicated in tumor development and lymph node metastasis in TC patients.

### Role of PLAU in immune cell infiltration

The association between PLAU expression and immune cell infiltration was also explored. The spearman correlation analysis revealed that PLAU expression levels were significantly, moderately to strongly, and positively correlated with infiltration levels of B cells (*r* = 0.391, *p* = 4.42e-19), CD8+T cells (*r* = 0.213, *p* = 2.16e-06), CD4+T cells (*r* = 0.361, *p* = 1.74e-16), macrophages (*r* = 0.273, *p* = 1.68e-10), neutrophils (*r* = 0.607, p = 1.68e-50), and dendritic cells (*r* = 0.634, p = 7.28e-56) (**Supplementary Figure S2F**). The SCNA module indicated that infiltrated levels of B cell, CD4+T cell, CD8+T cell, and macrophage were reduced in TC with arm-level deletion of PLAU compared to those with diploid/normal expression of PLAU. Conversely, neutrophils and dendritic cells were decreased in TC with arm-level deletion or arm-level gain of PLAU compared to those with diploid/normal expression of PLAU (**Supplementary Figure S2G**). According to the COX regression model of survival, the survival of TC patients was positively correlated with age (HR = 1.243, 95%CI, 1.124-1.373; *p* < 0.001), stage 4 (HR = 41.686, 95%CI, 2.669-650.993; *p* = 0.008), and dendritic cells (HR = 2.734e+07, 95%CI, 2.396-3.120e+11; *p* = 0.037), while it was negatively correlated with CD8+T cell (HR = 0, 95%CI, 0-0; *p* = 0.001), macrophage (HR = 0, 95%CI, 0-0.005; *p* = 0.031), and PLAU expression (HR = 0.636, 95%CI, 0.413-0.977; *p* = 0.039) as shown in **Supplementary Figure S2H**.

### PLAU promotes BA-induced suppression in KTC-1 cells with apoptosis and Golgi apparatus reprogramming

**Figure 3:**
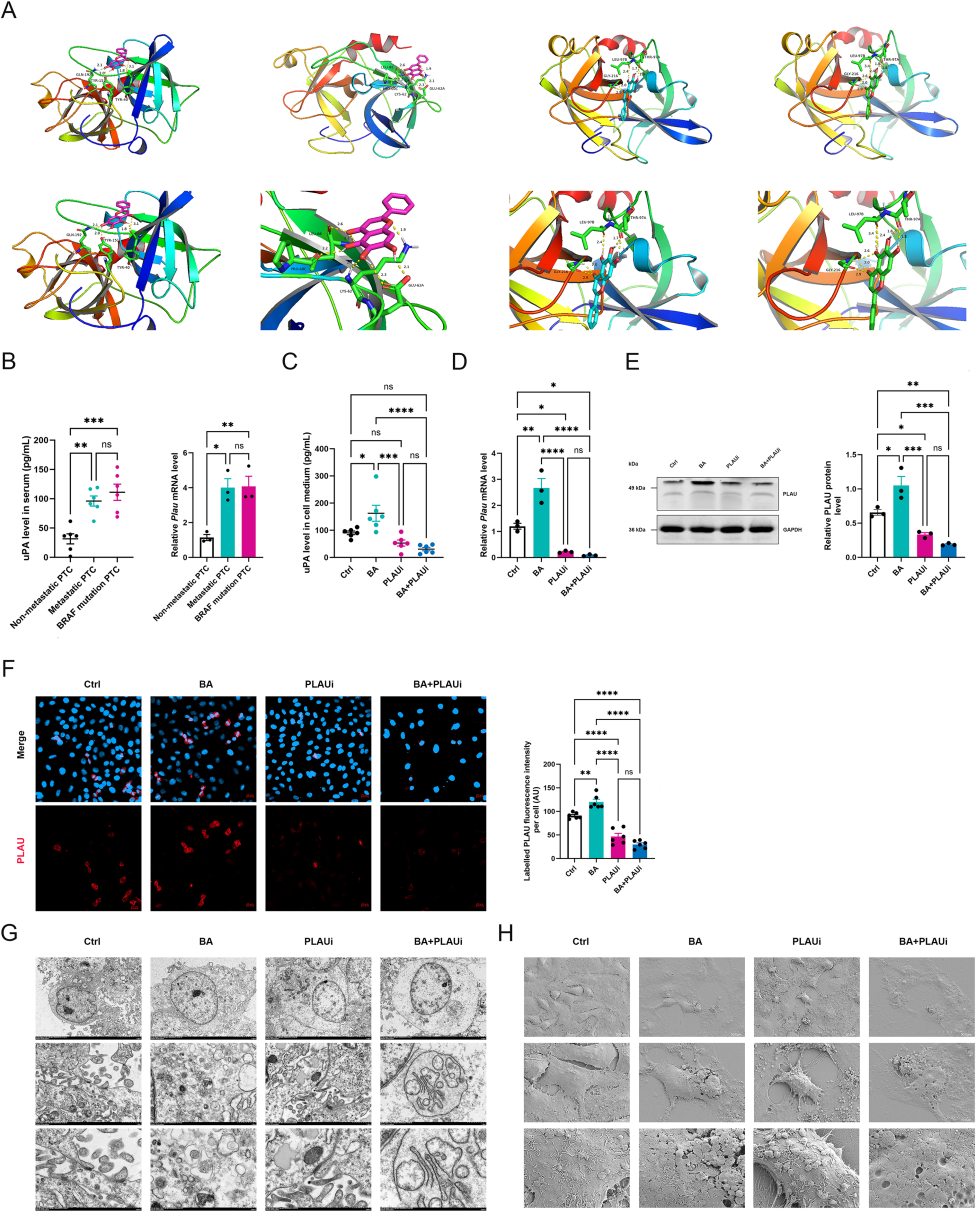
PLAU facilitates baicalein-induced suppression in KTC-1 cells. **(A)** Molecular docking was employed to simulate the interaction between baicalein and PLAU. **(B)** Serum levels of urokinase-type plasminogen activator and *Plau* mRNA expression in cancerous tissues from patients with papillary thyroid carcinoma comparing those without metastasis to those with metastasis or BRAF mutation. **(C)** Levels of urokinase-type plasminogen activator in the supernatant of the culture medium. **(D)***Plau* mRNA expression in KTC-1 cells. **(E)** Densitometric quantification of the PLAU/GAPDH protein ratio. **(F)** Immunofluorescent staining and quantitative analysis of PLAU expression in KTC-1 cells. Scale bar, 20 μm. **(G)** Representative transmission electron microscope images of KTC-1 cells. Scale bar, 5.0 μm, 1.0 μm, or 0.5 μm. **(H)** Representative scanning electron microscope images of KTC-1 cells. Scale bar, 50 μm, 10 μm, or 5 μm. Ctrl, control group with DMSO; BA, baicalein 100 μM; PLAUi, PLAU inhibitor (BC-11 hydrobromide); BA+PLAUi, combined treatment of baicalein 100 μM and PLAU inhibitor (BC-11 hydrobromide). All data are presented as mean ± S.E.M and analyzed by a one-way ANOVA with Turkey *t* test. All images is representative of three experiments. ns, not statistically; *P < 0.05; **P < 0.01; ***P < 0.001; ****P < 0.0001.

Molecular docking was employed to identify the potential targets to elucidate the molecular mechanism of BA against thyroid cancer. Our findings revealed four robust interactions between BA and PLAU protein, characterized by binding energies of -5.69, -5.38, -5.35, and -5.27 kcal/mol (**Figure 3A**, **Supplementary Table S2**). The serum levels of uPA protein encoded by PLAU and *Plau* mRNA were both increased in PTC patients with metastasis and BRAF mutation in comparison with those without metastasis. There were no statistical differences observed on serum uPA and *Plau* mRNA levels between DTC patients with metastasis and DTC patients BRAF mutation (**Figure 3B**).

To examine the impact of PLAU on tumor suppression mediated by BA, KTC-1 cells were subjected to treatment with DMSO, BA (100 μM), PLAUi (10 μM), and a combination of BA (100 μM) with PLAUi (10 μM). The BA treatment resulted in a higher serum uPA level compared to the other three groups (**Figure 3C**). Furthermore, it elevated the expression of *Plau* mRNA and PLAU protein expression relative to the PLAUi and BA+PLAUi groups. The levels of *Plau* mRNA levels and PLAU protein expression were progressively reduced in the Ctrl, PLAUi, and BA+PLAUi groups (**Figures 3D, E**). Correspondingly, alterations in labeled PLAU fluorescence intensity across these four groups paralleled levels of *Plau* mRNA (**Figure 3F**). The administration of BA alone enhances the expression of PLAU mRNA and protein, whereas the combination of BA with PLAUi markedly suppresses the PLAU expression relative to the other three groups.

Compared with Ctrl group, the KTC-1 cells in BA group exhibited nuclear swelling, an increase in apoptotic bodies and autophagosomes, and a reduction in extracellular vesicles. Treatment with PLAUi in KTC-1 cells resulted in chromatin margination, Golgi apparatus swelling, and an increase in lysosomes. However, the combined administration of BA and PLAUi led to nuclear swelling, chromatin margination, significant Golgi apparatus swelling or reprogramming with decrease in extracellular vesicles and lysosomes (**Figure 3G**). Scanning electron microscope revealed that KTC-1 cells treated with BA appeared shrunk with fewer protrusions or pseudopodia, flat and flattened bubbly structures. In contrast, KTC-1 cells exposed to PLAUi were swollen with increased protrusions, pseudopodia, and bubbly structures. Furthermore, cells treated with both BA and PLAUi were notably shrunk with even fewer protrusions or pseudopodia, more flattened bubbly structures, and some concavities (**Figure 3H**). These findings suggested that BA led to tumor suppression and apoptosis, while PLAUi enhanced vitality and invasive potential of KTC-1 cells. The combination treatment with BA and PLAUi demonstrated significant tumor suppression, accompanied by Golgi apparatus reprogramming.

### BA-mediated PLAU activation is associated with tumor apoptosis and migration in KTC-1 cells

**Figure 4:**
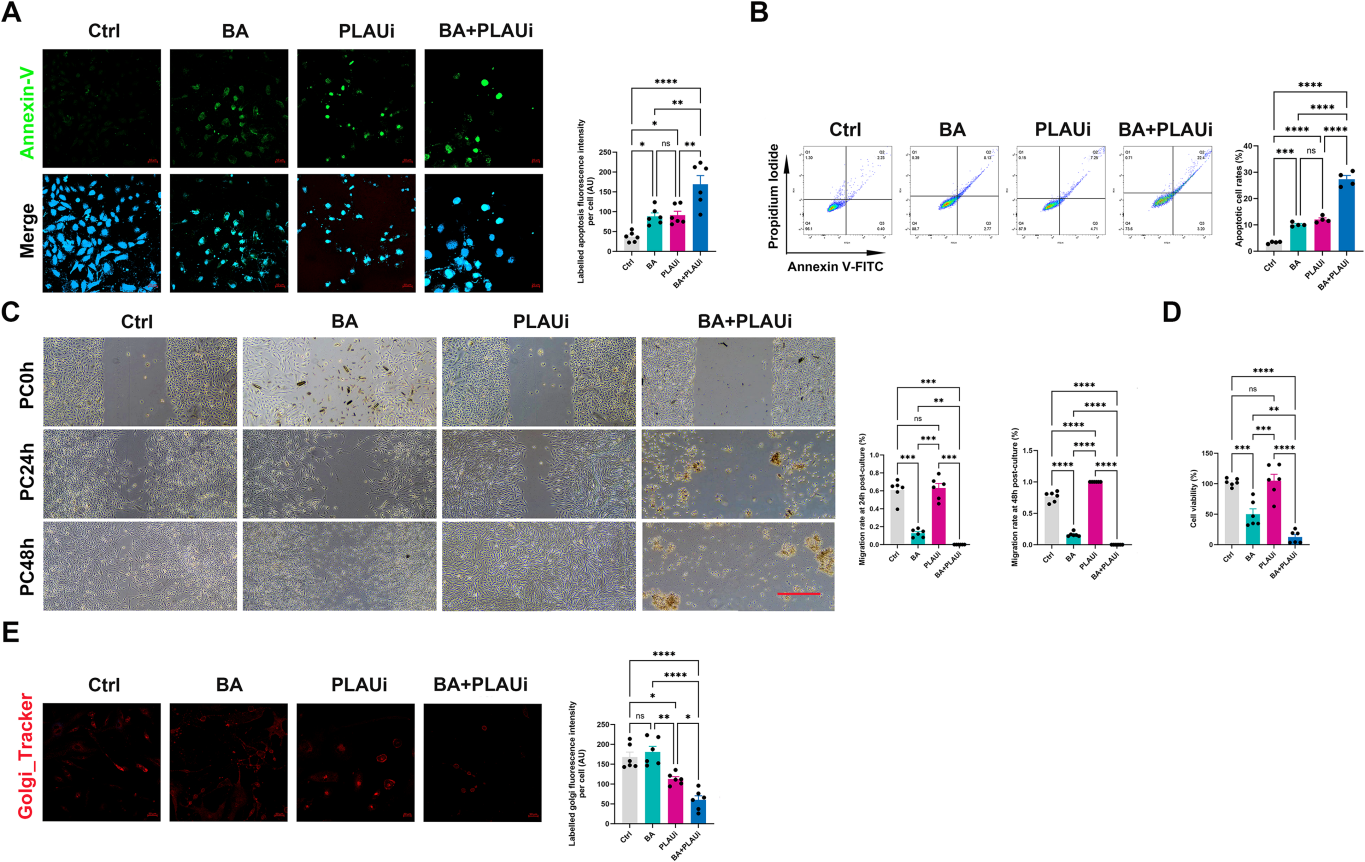
PLAU is associated with the suppression of biological behavior and the reprogramming of Golgi apparatus in KTC-1 cells by baicalein. **(A)** Immunofluorescent staining and quantitative analysis of apoptotic KTC-1 cells. Scale bar, 20 μm. **(B)** Cellular apoptosis was assessed using flow cytometry analyses. **(C)** Cell migration was assessed via cell scratch assays. Scale bar, 200 μm. **(D)** Cell viability was determined using a cell counting kit-8. **(E)** Immunofluorescent staining and quantitative analysis the of Golgi apparatus in KTC-1 cells. Scale bar, 20 μm. Ctrl, control group with DMSO; BA, baicalein 100 μM; PLAUi, PLAU inhibitor (BC-11 hydrobromide); BA+PLAUi, combined treatment of baicalein 100 μM and PLAU inhibitor (BC-11 hydrobromide). All data are presented as mean ± S.E.M and analyzed by a one-way ANOVA with Turkey *t* test. All images is representative of three experiments. ns, not statistically; *P < 0.05; **P < 0.01; ***P < 0.001; ****P < 0.0001.

The role of BA-mediated PLAU activation was further examined and intensity of apoptosis and apoptotic cell rates were elevated in the BA, PLAUi, and BA+PLAUi groups. However, combined treatment with BA and PLAUi resulted in significantly higher apoptotic fluorescence intensity and apoptotic cell rates compared to the BA and PLAUi groups individually, although no statistical difference was observed between these two groups (**Figures 4A, B**). Migration rates were markedly reduced in the BA group compared to the Ctrl and PLAUi groups at both 24 h and 48 h post-culture, with further inhibition seen in the BA+PLAUi group at the same point. However, the migration rate increased only in the PLAUi group relative to the Ctrl group at 48 h post-culture (**Figure 4C**). The changes in cell viability revealed the migration rates at 24 h post-culture (**Figure 4D**). Using the Golgi apparatus tracker to observe differences on Golgi apparatus reprogramming, the fluorescence intensities of the labelled Golgi apparatus were both lower in the PLAUi and BA+PLAUi groups as compared to Ctrl and BA groups. The fluorescence intensity of labeled Golgi apparatus was decreased in the BA+PLAUi group as compared to other groups, while no significant statistical difference was found between BA and Ctrl groups (**Figure 4E**). Moreover, fluorescence intensities of labeled mitochondria were significantly lower in BA, PLAUi, and BA+PLAUi groups than in Ctrl group, with BA+PLAUi group showing a decrease only in comparison to the BA group (**Supplementary Figures S3A, B**). In KTC-1 cells, BA significantly enhances apoptosis induction and inhibits migration, PLAUi also promotes apoptosis but favors migration, and combination of BA and PLAUi further induces apoptosis and suppressed migration, along with inhibition of PLAU-mediated Golgi apparatus reprogramming.

### BA modulates PLAU expression via inhibiting TPL2/MEK2/ERK2 pathway to regulate Golgi apparatus reprogramming

**Figure 5:**
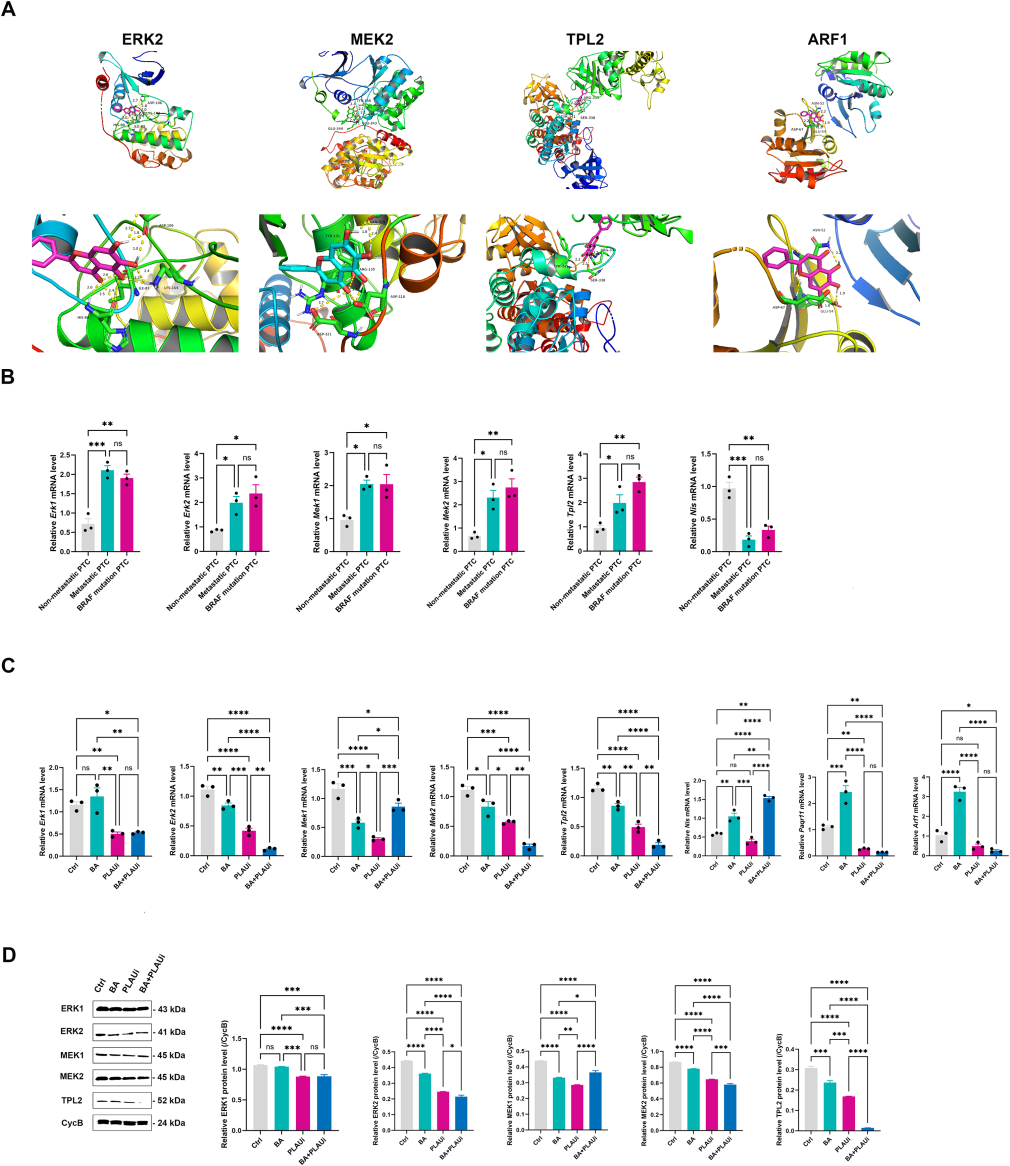
Inhibition of PLAU enhances the suppression of the MAPK pathway and Golgi apparatus reprogramming by baicalein in KTC-1 cells. **(A)** Molecular docking was employed to simulate the interaction between baicalein and key proteins such as ERK2, MEK2, TPL2, and ARF1. **(B)** qRT-PCR analysis was conducted to assess the expression levels of *Erk1*, *Erk2*, *Mek1*, *Mek2*, *TPL2*, and *Nis* mRNA expression in cancer tissues from patients with papillary thyroid carcinoma, comparing those without metastasis to those with metastasis or BRAF mutation. **(C)** qRT-PCR analysis of the mRNA expression of *Erk1*, *Erk2*, *Mek1*, *Mek2*, *Tpl2*, *Nis, Arf1, and Paqr11* in KTC-1 cells. **(D)** Relative protein expression of ERK1, ERK2, MEK1, MEK2, and TPL2. Ctrl, control group with DMSO; BA, baicalein 100 μM; PLAUi, PLAU inhibitor (BC-11 hydrobromide); BA+PLAUi, combined treatment of baicalein 100 μM and PLAU inhibitor (BC-11 hydrobromide). All data are presented as mean ± S.E.M and analyzed by a one-way ANOVA with Turkey *t* test. All images is representative of three experiments. ns, not statistically; *P < 0.05; **P < 0.01; ***P < 0.001; ****P < 0.0001.

The significance of classic signaling pathways, including PI3K/AKT/mTOR, MAPK, Wnt/β-catenin, JAK/STAT3 has been underscored as key targets through which BA exerting its anti-cancer effects (20). The loss of p53 triggers the formation of premetastatic secretory vesicles within Golgi apparatus, accompanied by an increase in expression of the Golgi apparatus scaffolding the protein-progestin and adipoQ receptor 11 (PAQR11). This protein recruits the complex containing adenosine the diphosphate ribosylation factor 1 (ARF1), a process facilitated by the PLAU receptor or signal transducer and activator of transcription-3-dependent pathway (21). Our findings demonstrated the robust interactions between BA and key proteins in the pathway, including ERK2, MEK2, TPL2, and ARF1, with a binding energy of -6.88, -6.94, -5.85, and -5.95 kcal/mol, respectively (**Figure 5A** and **Supplementary File**: **Supplementary Table S3**). PPI networks and Spearman’s correlation analysis both indicate that PLAU is linked to the MAPK pathway and Golgi apparatus reprogramming (**Supplementary Figures S4A-C**).

The PTC patients with metastasis or BRAF mutation exhibited elevated levels of *Erk1*, *Erk2*, *Mek1*, *Mek2*, and *Tpl2* mRNA and protein along with reduced levels of *Nis* mRNA and protein. However, there were no statistically significant differences in the mRNA levels of these genes between PTC patients with metastasis and those with BRAF mutation (**Figure 5B**). The KTC-1 cells treated with PLAUi or BA+PLAUi exhibited reduced levels of ERK1 mRNA and protein compared to those in Ctrl and BA groups. The MEK1 mRNA and protein levels were decreased progressively in Ctrl, BA+PLAUi, BA, and PLAUi groups, with all exhibiting statistical significance. Similarly, ERK2, MEK2, and TPL2 mRNA and protein levels decreased progressively in Ctrl, BA, PLAUi, and BA+PLAUi groups, with all demonstrating statistical significance (**Figures 5C, D**). Interestingly, KTC-1 cells treated with BA+PLAUi exhibited a lower level of *Nis* mRNA than the Ctrl group. BA in significantly up regulated mRNA levels of *Arf1* and *Paqr11* in comparison with the other three groups, although there were no statistical differences in *Arf1* and *Paqr11* mRNA levels between the Ctrl, BA, and BA+PLAUi groups (**Figure 5C**).

### BA suppresses tumor growth in TC xenograft mouse model via the PLAU-mediated TPL2/MEK2/ERK2 pathway

**Figure 6:**
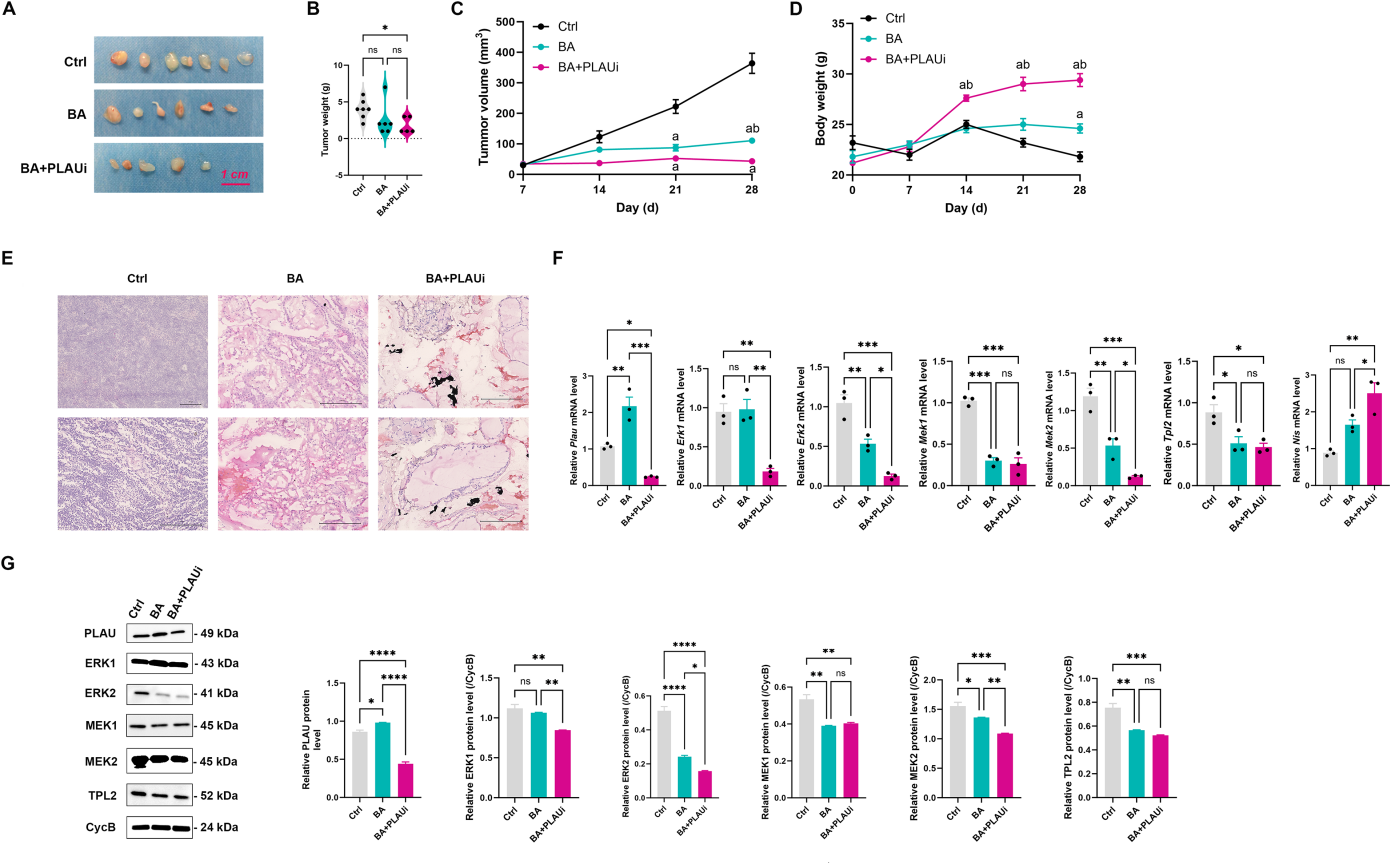
Inhibition of PLAU enhances the tumor growth suppression effect of baicalein in a thyroid cancer xenograft mouse model. **(A)** Treatment with baicalein resulted in a reduction in tumor size after 28 days (*n* = 5). **(B)** The combination of baicalein and PLAU inhibitor significantly decreased tumor weight. **(C)** The combination of baicalein and PLAU inhibitor significantly decreased tumor volumes. **(D)** Body weight fluctuations were tracked in all experimental groups over the study period. **(E)** Representative images of hematoxylin and eosin stain in the transplanted tumor tissues. **(F)** qRT-PCR analysis of the mRNA expression of *Plau, Erk1, Erk2, Mek1, Mek2, Tpl2, and Nis* in the transplanted tumor tissues. **(G)** Relative protein expression of PLAU, ERK1, ERK2, MEK1, MEK2, and TPL2 in the transplanted tumor tissues. Ctrl, control group with DMSO; BA, baicalein 100 μM; BA+PLAUi, combined treatment of baicalein 100 μM and PLAU inhibitor (BC-11 hydrobromide). For **(A)**, data are presented as Median with interquartile range and analyzed by a Brown-Forsythe and Welch ANOVA test with Dunnett T3 multiple comparison test. For **(C, D)**, data are presented as mean ± S.E.M and analyzed by a two way ANOVA test. For **(F, G)**, data are presented as mean ± S.E.M and analyzed by a one-way ANOVA with Turkey *t* test. All images is representative of three experiments. ns, not statistically; *P < 0.05; **P < 0.01; ***P < 0.001; ****P < 0.0001.

The inhibitory effect of BA on the growth of TC xenografts was further investigated. It was evident that tumors exposed to BA treatment were markedly reduced in size compared to those in control group. Furthermore, mice receiving both BA and PLAUi exhibited even smaller tumors than those treated solely with BA (**Figure 6A**). However, only xenografts mice treated with both BA and PLAUi exhibited a reduction in tumor weight compared to those in the control group (**Figure 6B**). Following three weeks of transplantation, xenografts mice treated with BA or a combination of BA and PLAUi demonstrated a lower tumor volume than those in the Ctrl group. At four weeks post-transplantation, the xenografts mice treated with both BA and PLAUi showed a decrease in the tumor weight compared to those in both the control and BA groups. Conversely, the tumor volume of the mice treated with BA alone was significantly greater than that of control group (**Figure 6C**). The average body weight of mice treated with BA and PLAUi was significantly greater than that of the Ctrl and BA groups (**Figure 6D**). In comparison to control group, tumors from mice treated with BA displayed a loosely organized and disordered architecture, along with an increased number of bubble-like structures. Additionally, treatment with both BA and PLAUi resulted in a diminished proliferation of cancer cells and a more disordered structure (**Figure 6E**).

BA could elevate the mRNA and protein level of PLAU, whereas co-administration of BA+PLAUi significantly reduced the mRNA and protein levels of the PLAU compared to Ctrl group. When comparing the BA+PLAUi group to control, both ERK1 mRNA and protein levels were diminished, and this reduction was also observed when comparing the BA+PLAUi group to BA group. However, there were no statistically significant differences in ERK1 mRNA and protein levels between the control and BA groups.

The mRNA and protein levels of ERK2 and MEK2 showed a progressive decline across control, BA, and BA+PLAUi groups. Conversely, mRNA and protein levels of MEK1 and TPL2 were reduced in both the BA and BA+PLAUi groups relative to Ctrl group, yet there were no significant differences between BA and BA+PLAUi groups. Additionally, the *Nis* mRNA level in the BA+PLAUi group was higher than in both Ctrl and BA groups (**Figures 6F, G**). Our results suggest that BA effectively suppresses the xenograft tumor growth, and combined administration of BA and PLAUi demonstrates a more potent anti-tumor effect, potentially through downregulation of TPL2/MEK2/ERK2 pathway.

## Discussion

PTC patients with regularly follow up the initial treatment are still facing the risk of developing advanced stage disease (22). These patients may experience extensive lymph node metastasis, resistance to RAI therapy, and ultimately progression to fatal diseases (5, 22). Approximately, 60% cases of PTC may harbor the *BRAF^V600E^* mutations, accompanied by aberrant activation of the MAPK pathway, molecular inhibition of the thyroid differentiation lineage, and remodeling of tumor immune microenvironment (23–25). Given the critical treatment landscape of PTC, there is a need for novel anticancer therapies with reduced adverse effects, stimulating research on novel natural sources of pharmacologically active compounds against PTC.

BA is a potential anti-cancer agent due to its effect on inhibiting growth, invasion, and metastasis of various tumor cells, as well as inducing the tumor cell apoptosis (26). Previous studies have reported the tumor inhibitory effects of BA against TC (16–18). Our previous studies have reported notable anti-TC effects of BA, which potentially enhances autophagy and apoptosis in TC cells, as well as induces cell cycle arrest through the activation of NF-κB signaling pathway (18). This study demonstrated that BA exerts growth-inhibitory effects on PTC *in vivo* and *in vitro* with increased apoptosis and suppressed migration.

*PLAU* facilitates cancer cell migration and invasion by encoding urokinase type plasminogen activator (uPA) (27, 28). Previous studies reported that PLAU expression levels are significantly elevated in the most cancer tissues, offering a certain degree of precision in diagnosing of different cancers (29). The overexpression of *PLAU* enhances the non-small cell lung cancer cell growth, survival, and cisplatin resistance by interacting with TM4SF1 to activate Akt signaling pathway (30). In *BRAF^V600E^* mutant TC cells, PLAU mediates tumor adhesion, invasion, and metastasis by activating Rel/MAP3K14/NF-κB pathway (31, 32). In this study, RNA-sequence, molecular docking, and *in vitro* experiments collectively demonstrated that BA significantly up-regulated *PLAU* expression, which is linked to the tumor progression, lymph node metastasis, and the infiltration of neutrophils and dendritic cells in TC.

Golgi apparatus reprogramming plays a pivotal role in the progression of cancer. Cancer cells can manipulate the Golgi apparatus’s defects in membrane trafficking to trigger signal transduction, proliferation, invasion, immune modulation, angiogenesis, and metastasis (33). The PLAU/PLAUR signaling pathway activates the PAQR11, which mediated the reorganization of cytoskeleton within the Golgi apparatus, facilitating the secretion of pre-metastatic effector protein PLOD3. Pharmacological inhibition of PLAUR has been shown to suppress the growth and metastasis of p53 deficient tumors (21). Targeting the Golgi apparatus to specifically disrupt its protein trafficking could induce apoptosis, presenting a potential strategy for effective cancer therapy (34, 35). This study found that BA induces tumor suppression and apoptosis by up-regulating PLAU expression and linked to Golgi apparatus reprogramming.

Hyper-activation of the MAPK signaling pathway to the *BRAF^V600E^* hotspot mutation occurs in aggressive TC, which induces the poor prognosis and limited therapeutic arsenal (36). To induce redifferentiation in refractory tumors, clinicians have explored the use of radioactive iodine therapy, which relies on the re-expression of the NIS (11, 25, 36, 37). Furthermore, Golgi protein 73 has been found to competitively bind with HECTD1, stabilizing growth factor receptor-bound protein 2 and thereby activating MAPK signaling pathway. This suggests that the Golgi apparatus plays a role in the activation of the MAPK pathway (38). The translocation of G protein βγ (Gβγ) to the Golgi apparatus, along with the activation and localization of ARF1, an effector downstream of Gβγ-PI3Kγ, and involved in the spatiotemporal regulation of G protein-coupled receptor signaling to MAPK (39–41). Molecular docking and biological experiments have revealed that BA down-regulates and interacts with proteins of the MAPK signaling pathway and ARF1 protein. The combined treatment of BA with PLAUi demonstrated a more potent inhibition of PLAU-mediated Golgi apparatus reprogramming and a stronger down-regulation of MAPK signaling pathway proteins.

## Conclusion

This study revealed the concurrent administration of BA and PLAUi could effectively suppressed the *PLAU* activation within PTC cells, leading to significant reduction in the transcription of *ARF1* and *PAQR11*. It further impedes the reprogramming of Golgi apparatus, ultimately promoting targeted inhibition of the TPL2/MEK2/ERK2 signaling pathway. Consequently, the inhibition of PLAU-mediated Golgi apparatus reprogramming and the blockade of the MAPK signaling pathway could present a promising therapeutic strategy for management of advanced thyroid cancer.

## Data Availability

The original contributions presented in the study are included in the article/**Supplementary Material**. Further inquiries can be directed to the corresponding author.

## Supplementary material

Supplementary Figure 1 Gene set enrichment analysis (GSEA) and pathway enrichment analysis of baicalein-treated KTC-1 cells. **(A)** GSEA of baicalein-treated KTC-1 cells. **(B)** Pathway enrichment analysis of baicalein-treated KTC-1 cells based on gene expression. The groups Ctrl, BA50, BA100, and BA200 represent KTC-1 cells treated with 0, 50, 100, and 200 μM baicalein, respectively.

Supplementary Figure 2 The correlation between PLAU expression and tumor subgroups along with immune cell infiltration. **(A)** The relative expression levels of PLAU in normal tissues and tumor samples. **(B)** The relative expression of PLAU in individuals with normal tissues or those diagnosed with TC, stratified by gender, race, and age. **(C)** The relative expression of PLAU in individuals with normal tissues or TC patients, categorized by pathological stage. **(D)** The relative expression of PLAU in individuals with normal tissues or TC patients, classified by tumor histology. **(E)** The relative expression of PLAU in individuals with normal tissues or TC patients, differentiated by the presence of lymph node metastasis. **(F)** Spearman’s correlation analysis illustrating the relationship between PLAU expression and various immune cell types. **(G)** SCNA analysis examining the infiltration levels of immune cells in diploid/normal samples, samples with arm-level deletion, or samples with arm-level gain of PLAU. **(H)** Outcomes and forest plot from the COX regression model for survival analysis. HR, hazard ratio. **P* < 0.05; ***P* < 0.01; ****P* < 0.001; *****P* < 0.0001.

Supplementary Figure 3 Labeled mitochondria in baicalein-treated KTC-1 cells. **(A)** Immunofluorescent staining of mitochondira in KTC-1 cells. Scale bar, 50 μm. **(B)** Immunofluorescent staining and quantitative analysis of mitochondira in KTC-1 cells. Scale bar, 20 μm.Ctrl, control group with DMSO; BA, baicalein 100 μM; PLAUi, PLAU inhibitor (BC-11 hydrobromide); BA+PLAUi, combined treatment of baicalein 100 μM and PLAU inhibitor (BC-11 hydrobromide). All data are presented as mean ± S.E.M and analyzed by a one-way ANOVA with Turkey *t* test. All images is representative of three experiments. ns, not statistically; **P* < 0.05; ***P* < 0.01; ****P* < 0.001; *****P* < 0.0001.

Supplementary Figure 4 The relationship between PLAU expression and MAPK pathway. **(A)** The network of interactions between PLAU and proteins in the MAPK pathway. **(B)** The protein-protein interaction network involving PLAU and proteins of the MAPK pathway. **(C)** Spearman’s correlation analysis examining the relationship between PLAU expression and proteins of the MAPK pathway, as well as ARF1 and PAQR11 (MMD). **(D)** Heatmap illustrating the expression levels of PLAU, ERK1, ERK2, MEK1, and MEK2 in KTC-1 cells treated with baicalein.

Supplementary Table 1 Shows the list of primary antibodies used in this study.

Supplementary Table 2 Details of molecular docking between BA and PLAU.

Supplementary Table 3 Details of molecular docking between BA and target proteins.
