# Supplementary material for: Baicalein inhibits the progression of thyroid cancer by suppressing the TPL2/MEK2/ERK2 pathway
Source: Front Endocrinol (Lausanne). 2026 Jan 28;17:1739944. doi: 10.3389/fendo.2026.1739944 (PMC12890616; doi:10.3389/fendo.2026.1739944)
Supplement: Supplementary Figure 1 — Gene set enrichment analysis (GSEA) and pathway enrichment analysis of baicalein-treated KTC-1 cells. (A) GSEA of baicalein-treated KTC-1 cells. (B) Pathway enrichment analysis of baicalein-treated KTC-1 cells based on gene expression. The groups Ctrl, BA50, BA100, and BA200 represent KTC-1 cells treated with 0, 50, 100, and 200 μM baicalein, respectively. [file DataSheet1.pdf]

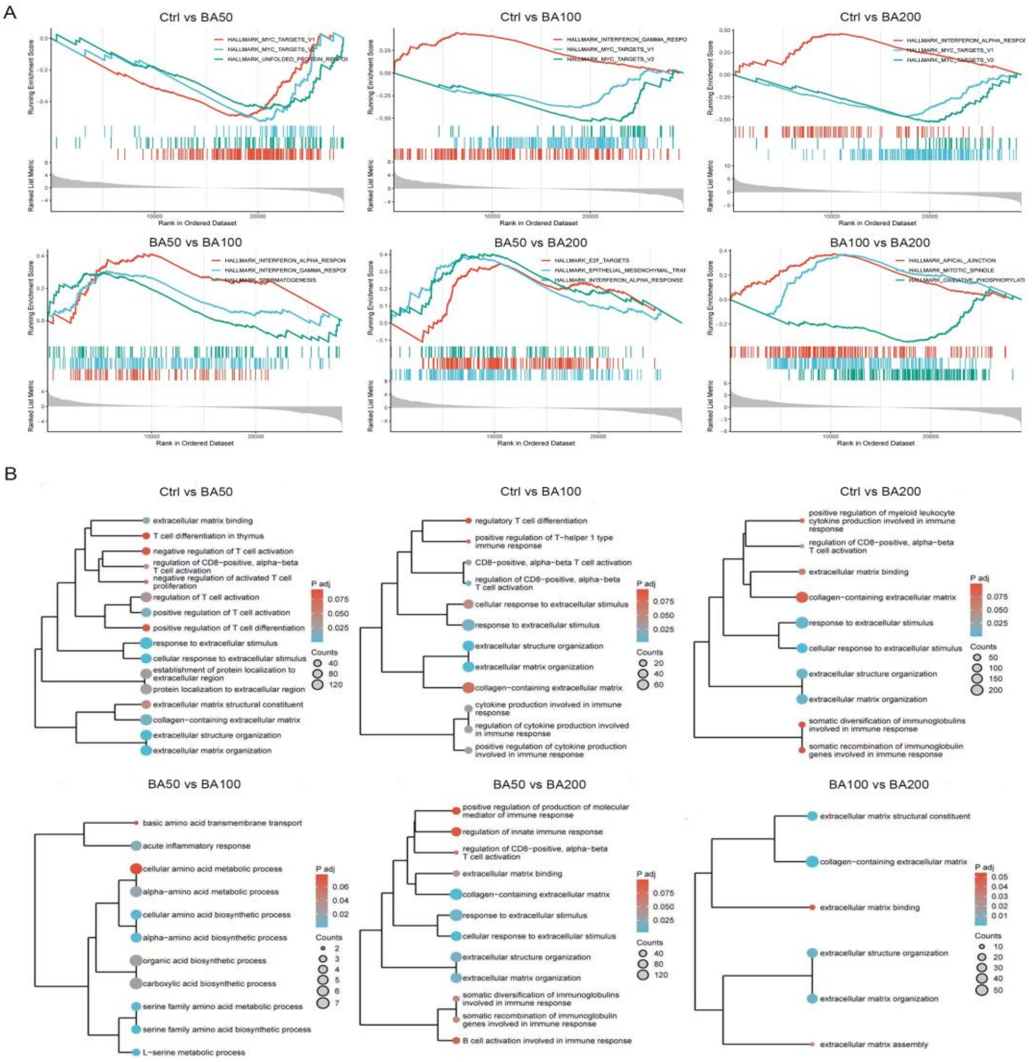

Figure S1: Gene set enrichment analysis (GSEA) and pathway enrichment analysis of bailcalein-treated KTC-1 cells. (A) GSEA of bailcalein-treated KTC-1 cells. (B) Pathway enrichment analysis of bailcalein-treated KTC-1 cells based on gene expression. The groups Ctrl, BA50, BA100, and BA200 represent KTC-1 cells treated with 0, 50, 100, and 200 μM bailcalein, respectively.

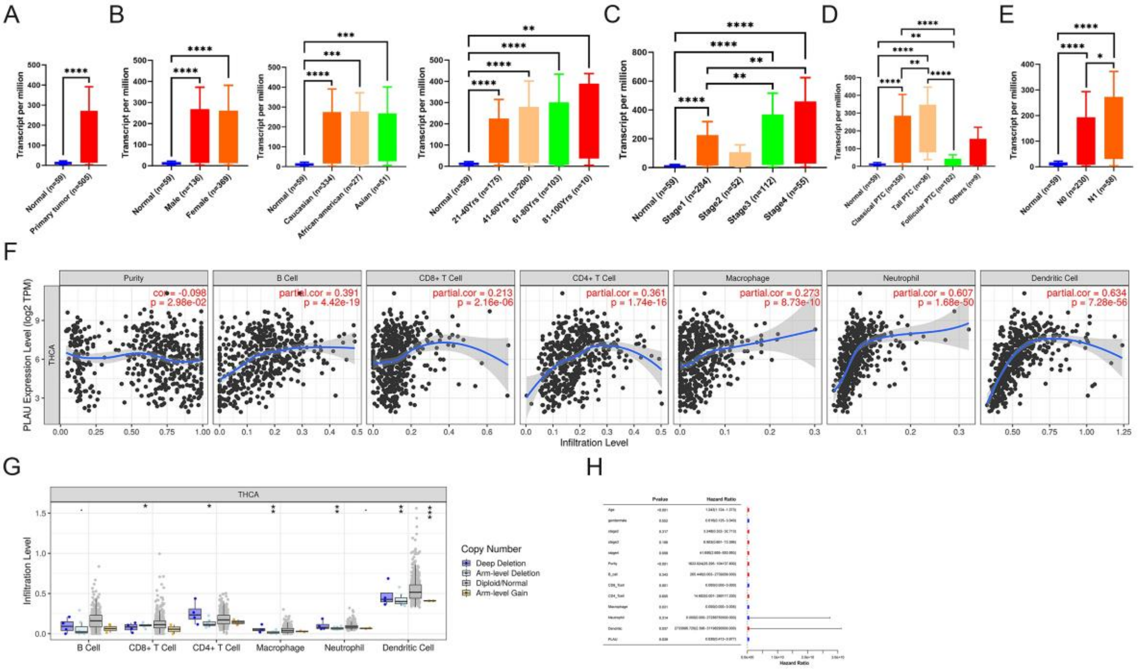

Figure S2: The Correlation Between PLAU Expression and Tumor Subgroups along with Immune Cell Infiltration. (A) The relative expression levels of PLAU in normal tissues and tumor samples. (B) The relative expression of PLAU in individuals with normal tissues or those diagnosed with TC, stratified by gender, race, and age. (C) The relative expression of PLAU in individuals with normal tissues or TC patients, categorized by pathological stage. (D) The relative expression of PLAU in individuals with normal tissues or TC patients, classified by tumor histology. (E) The relative expression of PLAU in individuals with normal tissues or TC patients, differentiated by the presence of lymph node metastasis. (F) Spearman's correlation analysis illustrating the relationship between PLAU expression and various immune cell types. (G) SCNA analysis examining the infiltration levels of immune cells in diploid/normal samples, samples with arm-level deletion, or samples with arm-level gain of PLAU. (H) Outcomes and forest plot from the COX regression model for survival analysis. HR, hazard ratio. *$P < 0.05$; **$P < 0.01$; ***$P < 0.001$; ****$P < 0.0001$.

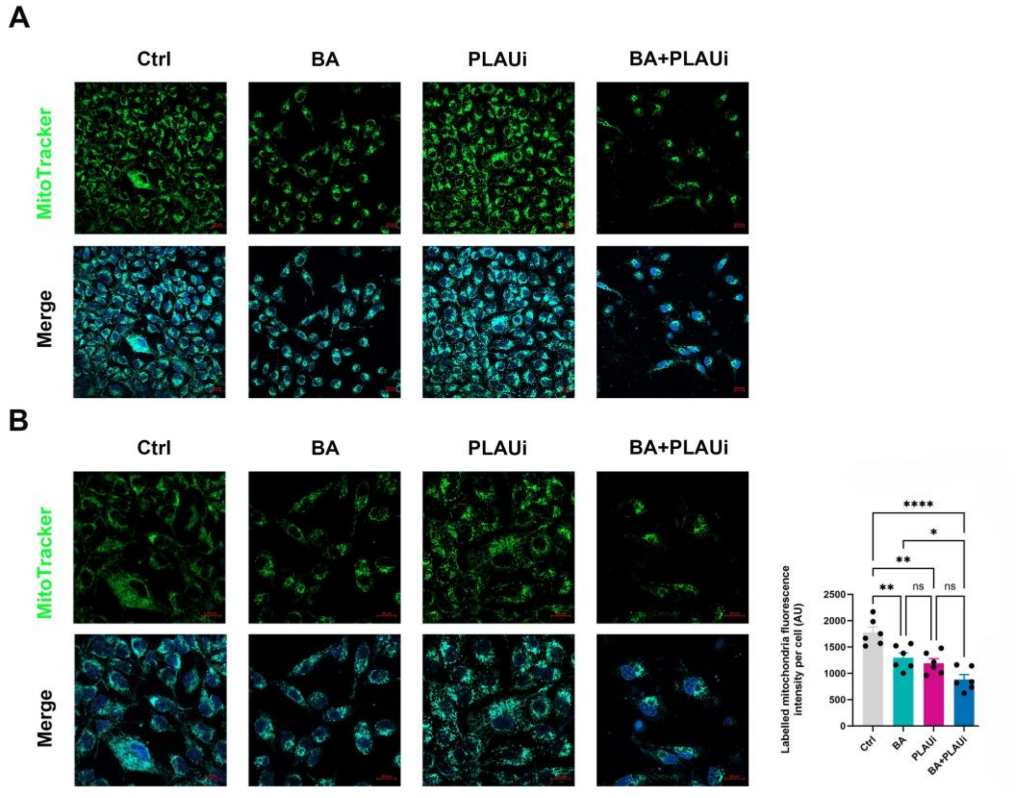

Figure S3: Labeled mitochondria in bailcalein-treated KTC-1 cells. (A) Immunofluorescent staining of mitochondira in KTC-1 cells. Scale bar, 50 μm. (B) Immunofluorescent staining and quantitative analysis of mitochondira in KTC-1 cells. Scale bar, 20 μm.Ctrl, control group with DMSO; BA, bailcalein 100 μM; PLAUi, PLAU inhibitor (BC-11 hydrobromide); BA+PLAUi, combined treatment of bailcalein 100 μM and PLAU inhibitor (BC-11 hydrobromide). All data are presented as mean ± S.E.M and analyzed by a one-way ANOVA with Turkey *t* test. All images is representative of three experiments. ns, not statistically; *P < 0.05; **P < 0.01; ***P < 0.001; ****P < 0.0001.

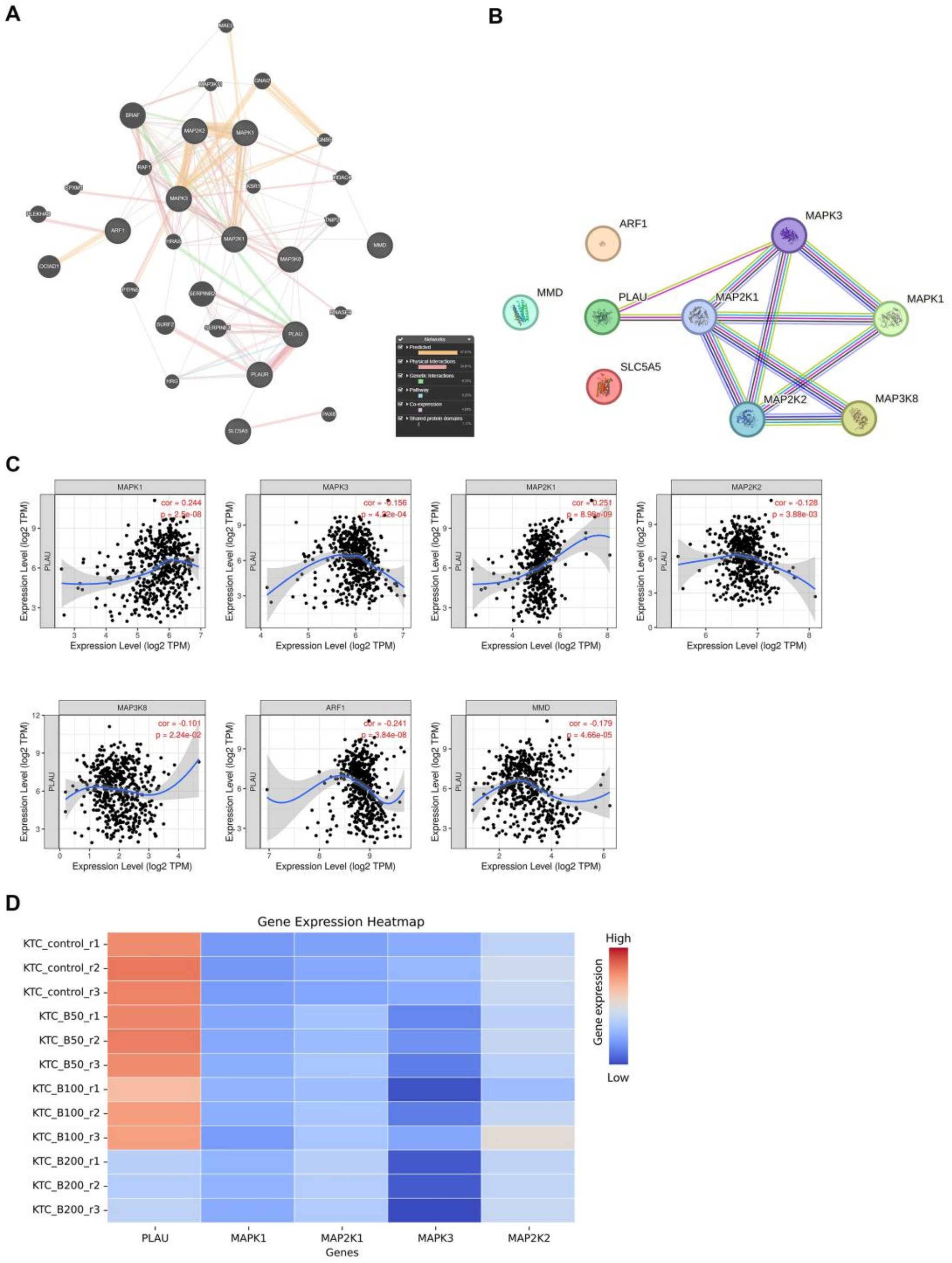

Figure S4: The relationship between PLAU expression and MAPK pathway. (A): The network of interactions between PLAU and proteins in the MAPK pathway. (B) The protein-protein interaction network involving PLAU and proteins of the MAPK pathway. (C) Spearman's correlation analysis examining the relationship between PLAU expression and proteins of the MAPK pathway, as well as ARF1 and PAQR11

(MMD). (D) Heatmap illustrating the expression levels of PLAU, ERK1, ERK2, MEK1, and MEK2 in KTC-1 cells treated with bailcalein.

Table S1: Shows the list of primary antibodies used in this study

Products	Catalog	Concentration	Manufacturer
Anti-Urokinase antibody[EPR6273]	ab133563	1/1000	Abcam
Anti-ERK1 antibody [Y72]	ab32537	1/1000	Abcam
Anti-ERK2 antibody [E460]	ab32081	1/1000	Abcam
Anti-MEK1 antibody [Y77]	ab32576	1/10000	Abcam
Anti-MEK2 antibody [Y78]	ab32517	1/3000	Abcam
Anti-MAP3K8/COT antibody [RP23040075]	ab308011	1/1000	Abcam
Anti-Cyclophilin B antibody [EPR12703(B)]-Loading Control	ab178397	1/1000	Abcam

Table S2: Details of molecular docking between BA and PLAU

Protein	Molecular	Model	B.E (kcal/mol)	A.A residues	H-bonds (Å)
PLAU	5YC6	5YC61	-5.69	GLN-192	2.1
				TYR-151	2.0
				TYR-40	3.1/1.8
		5YC62	-5.38	GLU-62A	2.3/2.1
				LYS-62	2.4/1.9
				PRO-60C	2.2
				LEU-88	2.6/2.6
		5YC63	-5.35	LEU-97B	2.4
				GLY-216	2.9/2.0
				THR-97A	1.9/1.7
		5YC64	-5.27	GLY-216	2.9/2.6/2.0
				LEU-97B	3.4/2.4
				THR-97A	1.8/1.8

Table S3: Details of molecular docking between BA and target proteins

Protein	Molecular	B.E (kcal/mol)	A.A residues	H-bonds (Å)
ERK1	4QTB1	-4.95	GLU-344	2.9/2.6
			GLU-343	2.6/2.1
			TYR-156	2.3/2.1
ERK2	8AOJ	-6.88	HIS-80	2.5/2.4/2.0
			ASP-106	2.7/2.0/1.8
			ILE-83	2.6
			LYS-164	2.4/2.2
MEK1	3DV3	-5.67	ASP-370	2.0/1.9

			ALA-372	2.0
			PHE-371	2.0
MEK2	4H3Q	-6.94	GLN-315	2.4/1.8
			TYR-131	3.5
			ARG-135	1.8/1.8
			ASP-318	2.9/1.5
			ASP-321	3.7
TPL2	4Y85	-5.85	ARG-333	2.5
			TYR-341	2.2
			SER-338	2.1
ARF1	1HUR	-5.95	ASN-52	2.2
			ASP-67	2.2
			GLU-54	1.9/1.8
